# Gut–Liver Axis Dysregulation in Portal Hypertension: Emerging Frontiers

**DOI:** 10.3390/nu16071025

**Published:** 2024-04-01

**Authors:** Martina Lombardi, Jacopo Troisi, Benedetta Maria Motta, Pietro Torre, Mario Masarone, Marcello Persico

**Affiliations:** 1 Department of Chemistry and Biology “A. Zambelli”, University of Salerno, Via Giovanni Paolo II 132, 84084 Fisciano, SA, Italy; jtroisi@unisa.it; 2 European Institute of Metabolomics (EIM) Foundation, Via G. Puccini, 3, 84081 Baronissi, SA, Italy; 3 Department of Medicine, Surgery and Dentistry, “Scuola Medica Salernitana”, University of Salerno, 84081 Baronissi, SA, Italy; bmotta@unisa.it (B.M.M.); ptorre@unisa.it (P.T.); mmasarone@unisa.it (M.M.)

**Keywords:** gut–liver axis, portal hypertension, microbiome, metabolomics, liver diseases

## Abstract

Portal hypertension (PH) is a complex clinical challenge with severe complications, including variceal bleeding, ascites, hepatic encephalopathy, and hepatorenal syndrome. The gut microbiota (GM) and its interconnectedness with human health have emerged as a captivating field of research. This review explores the intricate connections between the gut and the liver, aiming to elucidate how alterations in GM, intestinal barrier function, and gut-derived molecules impact the development and progression of PH. A systematic literature search, following PRISMA guidelines, identified 12 original articles that suggest a relationship between GM, the gut–liver axis, and PH. Mechanisms such as dysbiosis, bacterial translocation, altered microbial structure, and inflammation appear to orchestrate this relationship. One notable study highlights the pivotal role of the farnesoid X receptor axis in regulating the interplay between the gut and liver and proposes it as a promising therapeutic target. Fecal transplantation experiments further emphasize the pathogenic significance of the GM in modulating liver maladies, including PH. Recent advancements in metagenomics and metabolomics have expanded our understanding of the GM’s role in human ailments. The review suggests that addressing the unmet need of identifying gut–liver axis-related metabolic and molecular pathways holds potential for elucidating pathogenesis and directing novel therapeutic interventions.

## 1. Introduction

### 1.1. Gut–Liver Axis

The human gut microbiome (GM) frequently denoted as the “forgotten organ”, is a dynamic and densely populated community of microorganisms residing within the gastrointestinal tract. Comprising approximately 100 trillion microbial cells, including bacteria, viruses, fungi, eukarya, and archaea, the GM exerts a profound influence over numerous physiological processes, extending far beyond the confines of digestion [1,2]. Indeed, its intricate interactions with the host organism contribute to the maturation and modulation of the immune system, the extraction of nutrients from dietary sources, the synthesis of vitamins and bioactive compounds, as well as the protection against pathogenic invaders [3]. Furthermore, recent research has unveiled the GM’s role in metabolic regulation, influencing energy extraction from food, insulin sensitivity, and lipid metabolism [4]. Dysregulation of this finely tuned metabolic orchestra has been linked to diverse pathological conditions including obesity, diabetes, and metabolic syndrome, and has even been associated with mental health and neurological conditions [5].

The gut–liver axis (GLA), a fundamental concept in human physiology, denotes a bidirectional relationship between the gastrointestinal system, specifically the GM, and the liver. This intricate interaction arises from the complex interplay of signals generated by various factors, including dietary choices, genetic predisposition, and environmental elements. The paramount significance of this relationship becomes evident in understanding the consequences of disruptions in the intestinal barrier, which lead to an enhanced flow of bacteria and their byproducts into the liver. These perturbations in the GLA can trigger or exacerbate a broad range of hepatic ailments, underscoring the interdependence of these vital organs. While the role of the GM in liver disorders, such as alcohol-related liver disease (ALD) and bacterial infections associated with advanced liver disease, has been appreciated for some time [6,7], the pivotal significance of a perturbed GLA in the pathogenesis of numerous liver diseases has gained recognition in recent times. This newfound acknowledgment has been facilitated by the progressive accumulation of knowledge concerning the composition and functions of the GM, the maintenance of the intestinal barrier, and the role of bile in mediating communication between the gut and the liver.

The intricate reciprocity between the GM and the liver is established through the portal vein, a vital conduit responsible for transporting substances derived from the gut to the liver. On the other hand, the liver sends back bile and antibodies to the intestine. Beyond its role in regulating metabolic functions through nuclear receptors, bile acids assume a pivotal position in governing the composition of the GM. The essential point of exchange between the liver and the GM occurs at the gut mucosal barrier, that constitutes a multifaceted system composed of intestinal epithelial cells. These specialized cells serve as the guardians of gut homeostasis, acting as a physical barricade that keeps the GM segregated from host immune cells.

A cornerstone of this defensive architecture is the mucus barrier, a specialized structure that effectively segregates the GM from the epithelial lining, functioning as a bulwark against unwarranted inflammatory responses. In rare instances, some microbes, like segmented filamentous bacteria, primarily encountered in early human life [8], can breach the mucus barrier and engage in host-specific interactions. However, for the majority of microbial residents, their interaction with the host takes an indirect route, primarily mediated by the release of metabolic products known as postbiotics, generated during food fermentation [9,10,11]. The thickness of the mucus barrier changes throughout the gastrointestinal tract, reaching its zenith in regions hosting a substantial microbiota population, like terminal ileum and colon [12]. Within the latter, the mucus barrier adopts a stratified structure, with two different layers: an inner one, which closely interfaces with the epithelium; and an outer layer colonized by various bacterial species [12]. Some microbial strains can anchor themselves to this outer mucus layer, thereby avoiding displacement by the contractile movements of peristalsis. Microbes unable to adhere to the mucus layer can gain access by way of mucin-IgA interactions [13]. The inner mucus layer remains nearly sterile, owing to its constrictive mesh size and the presence of antimicrobial peptides [14] and microbiota-excluding proteins like lypd8 and ZG16 [15]. These proteins form a robust line of defense, as they interact with numerous bacterial groups, obstructing their penetration of the inner mucus layer. Intriguingly, the constitution of the mucus barrier can be significantly molded by the resident microbiota, as evidenced by experiments involving germ-free mice colonized with GM, leading to the development of mucus reminiscent of that of the donor mice [16]. This phenomenon can be attributed to goblet cells’ capacity to detect bacterial products, subsequently triggering the production of Muc2 through the activation of the NLRP6-inflammasome pathway [15]. Conversely, the mucus barrier also serves as a nutrient reservoir for numerous bacterial species, with *Akkermansia muciniphila* being a prime example, capable of metabolizing mucins for growth [17]. Pertinently, the absence of dietary fibers can lead to the overgrowth of mucin-degrading bacteria, compromising the thickness of the mucus layer [18]. Additionally, the balance between *Bacteroides* and *Firmicutes* holds the potential to influence the glycosylation of mucin, thereby affecting the composition of the microbiota. Such variations in mucosal structure, as observed in cases of inflammatory bowel disease, notably ulcerative colitis, can precipitate direct interactions between the GM and epithelium, ultimately playing a role in the initiation and perpetuation of inflammatory responses. Therefore, the mucus barrier emerges as a pivotal defense mechanism that also serves as nutrient reservoir and habitat facilitating microbiota colonization, thus preventing their expulsion through peristaltic movements.

Directly beneath the mucus layer, the gut barrier takes the form of a single layer of epithelial cells, each comprising enterocytes, goblet cells, tuft cells, and enterochromaffin cells [19]. These multifarious cells collaboratively function in protecting the gastrointestinal tract from potential threats posed by microbiota and infectious agents. This defensive structure manifests as a physical barrier, thanks to the tight junctions sealing adjacent epithelial cells, an electrical barrier, facilitated by the negative charge on the brush border counteracting the microbiota’s negative charge, and a chemical barrier, with epithelial cells releasing antimicrobial peptides. Furthermore, an array of mucosal immune cells patrols the epithelium. In addition, within the lamina propria plasma cells are plentiful, secreting immunoglobulin A (IgA) to augment the protective capabilities of the barrier. In the event that the epithelium is breached by bacteria, through the active mechanisms enacted by invasive pathogens or pathobionts or as a result of injury, bacteria may infiltrate the lamina propria. Nevertheless, just a small proportion of these bacteria succeeds in disseminating throughout the organism. A few of them may reach the mesenteric lymph nodes, functioning as a barrier to hinder microbes from infiltrating the systemic bloodstream. This safeguarding is accomplished through the presence of the gut vascular barrier [20,21], by which the passage of bacteria into the portal circulation and their access to the liver is prevented. Notably, some pathogenic bacteria and perhaps certain pathobionts have developed mechanisms to circumvent this defensive system. An example of this phenomenon can be observed in cases of Salmonella infection, where the disruption of this barrier leads to the systematic dissemination of the bacteria [21]. Remarkably, this barrier is compromised in certain pathological contexts such as metabolic steatohepatitis (MASH—previously referred to as non-alcoholic steatohepatitis, NASH) [22].

The functional capacity of the GM is arguably more pertinent than its composition when considering its impact on health. Components derived from bacteria, named pathogen-associated molecular patterns (PAMPs), as well as metabolites resulting from the actions of the GM on both exogenous (from diet and environmental exposure) and endogenous (bile acids and amino acids) substrates can reach the liver via the portal vein, where they have the potential to incite inflammation. The role of PAMPs in liver damage in metabolic-associated Steatotic liver disease (MASLD—previously named non-alcoholic fatty liver disease, NAFLD) is substantiated by preclinical investigations revealing an attenuation of hepatic steatosis, inflammation, and fibrosis in mice deficient in toll-like receptors 4 (TLR-4) or 9 (TLR-9) under conditions of a high-fat or choline-deficient diet [23,24,25]. Moreover, changes in the GM composition resulting from inflammasome deficiency have been found to induce hepatic steatosis and inflammation. This occurs by means of the portal inflow of TLR-4 and TLR-9 agonists in mice, leading to heightened hepatic tumor necrosis factor alpha (TNF-α) expression and inflammation, a phenomenon particularly pronounced in mouse models of hepatic steatosis [26]. Alterations in the composition of the GM can result in a gut imbalance, with lower bioavailability of choline and a rise in the portal afflux of trimethylamine, with both of them being linked to hepatic steatosis in both human and experimental models. The role of the GM in this context is highlighted by the capacity of certain microbes to metabolize choline, thereby reducing its bioavailability and generating various metabolites, including trimethylamine [27,28]. Moreover, an enhanced ethanol production ability has been observed in microbial populations of children suffering from MASH [29,30]. Furthermore, MASLD has been associated with GM-derived products of branched-chain and aromatic amino acid metabolism, including phenylacetic acid and 3-(4-hydroxyphenyl)lactate, both of which are linked to insulin resistance. A cohort of obese, non-diabetic patients with hepatic steatosis and inflammation, exhibited reduced diversity in microbial genes, a higher microbial genetic capacity to metabolize dietary lipids, increased production of endotoxins from *Proteobacteria*, and disrupted metabolism of aromatic and branched-chain amino acids [31]. Additionally, the potential pathogenic impact of metabolites derived from the GM is also supported by experiments where transplanting fecal microbiome from human donors with hepatic steatosis, as well as consistently administering phenylacetic acid, induced steatosis in mice. The production of short-chain fatty acids (SCFAs) via bacterial fermentation of indigestible carbohydrates (such as dietary fiber) serves as a prominent illustration of the mutually beneficial interaction between GM and host. Diet supplies non-digestible carbohydrates that nourish bacterial growth, while, in exchange, these microorganisms produce SCFAs, such as butyrate, which serve as an energy substrate for colonocytes, alleviate intestinal inflammation, and regulate satiety [32,33]. In obesity and MASLD, however, the connection between clinical characteristics and SCFAs remains incongruent and may be attributable to differences in the levels of individual SCFAs, each potentially exerting unique influences on the metabolism of the host. The GM of patients with type-2 diabetes mellitus is characterized by lower levels of butyrate-producing bacteria [34]. Supplementation with SCFAs ameliorates diet-induced hepatic steatosis in mice. Consistent with these findings, a pivotal randomized study demonstrated that a high-fiber diet in patients suffering from type-2 diabetes mellitus significantly affects GM fermentation of carbohydrates, promoting a wider diversity and abundance of butyrate-producing bacteria and improving hemoglobin A1c levels, partly through increased glucagon-like peptide-1 (GLP-1) production [35].

Impaired bile acid signaling is an additional outcome of the GM alterations observed in mice fed a high-fiber diet and in humans with MASLD. In these contexts, the GM seems to be characterized by an excess of bacteria that generate secondary bile acids, such as deoxycholic acid, which serves as an FXR antagonistic bile acid, suppressing FXR- and FGFR4-mediated signaling [36]. The consequence of this disruption is an increased synthesis of bile acids, leading to elevated serum concentrations of primary and secondary bile acids [36,37]. Thus, the composition of the GM influences the production of secondary bile acids and impacts FXR-mediated signaling in the intestine and the liver.

### 1.2. Portal Hypertension

The term “portal hypertension” (PH) was originally coined by Gilbert and Carnot in 1902 [38]. It refers to an elevation in pressure in the portal venous system, due to a heightened portal pressure gradient, which represents the difference in pressure between the portal venous system and hepatic vein or inferior vena cava. In normal circumstances, this gradient is equal to or less than 5 mmHg. However, a portal pressure gradient of 6 mmHg or greater between the portal and hepatic veins (or inferior vena cava) typically indicates the existence of PH, with a gradient exceeding 10 mmHg reaching “clinical significance” (i.e., clinically relevant signs and symptoms) [39]. The onset of PH is associated with a rise in resistance to portal blood flux, a phenomenon that can occur within the liver, as observed in cirrhosis, or external to the liver, such as in pre-hepatic conditions like portal vein thrombosis, or post-hepatic conditions like constrictive pericarditis or Budd–Chiari syndrome. Determining the level of resistance to portal blood flow aids in diagnosing the underlying cause of PH, a condition that ranks as the most common reason for hospitalization, variceal bleeding, liver transplantation, and mortality in individuals with cirrhosis.

PH’s causes can be classified into pre-hepatic, intrahepatic, or post-hepatic. Pre-hepatic etiologies often stem from either heightened blood flow or obstructions in the portal vein or splenic vein. Conditions leading to higher blood flow may encompass idiopathic tropical splenomegaly, arterio-venous malformations, or fistulas, while obstructions within the portal or splenic veins may be a result of thrombosis, infiltration, or compression of these veins by tumors. Intrahepatic causes can be further subcategorized as pre-sinusoidal, sinusoidal, or post-sinusoidal causes. Pre-sinusoidal intrahepatic factors can be attributed to schistosomiasis, congenital hepatic fibrosis, early primary biliary cholangitis, sarcoidosis, chronic active hepatitis, and exposure to toxins like vinyl chloride, arsenic, and copper. Sinusoidal causes are linked to cirrhosis, alcoholic hepatitis, vitamin A intoxication, or cytotoxic drugs. Post-sinusoidal causes are related to conditions such as sinusoidal obstruction syndrome or veno-occlusive disease [39].

Ultimately, post-hepatic causes may manifest at the cardiac level, the hepatic vein, like in Budd–Chiari syndrome, as well as at the inferior vena cava. Heart-related causes frequently stem from elevated atrial pressure, as seen in constrictive pericarditis. When these causes manifest at the inferior vena cava level, they are usually attributed to stenosis, thrombosis, the presence of webs, or tumor invasion [39].

In epidemiological terms, liver cirrhosis stands as the predominant cause of PH in the Western world, while schistosomiasis leads in the African continent, where the disease is endemic [39].

The pathophysiology of PH is closely tied to the portal venous system, which is formed as the superior mesenteric vein and splenic vein converge creating the portal vein. This vessel directs its flow into the liver before branching into the right and left portal veins, which supply blood to the respective lobes. Typically, the portal vein pressure is slightly higher (1 to 4 mmHg) than the hepatic vein pressure, allowing blood to flow through the liver and into the systemic circulation. Importantly, these veins lack valves. Resistance to the blood flow within the portal venous tract results in increased portal venous pressure, a hallmark of PH. This resistance can occur within the liver, as in cirrhosis, or at pre-hepatic or post-hepatic sites, and may be attributed to structural or dynamic changes. Structural alterations arise from modifications in hepatic microcirculation, which result from the activation of hepatic stellate cells and the subsequent development of fibrosis, regenerative nodules, vascular occlusion, and angiogenesis. Augmented levels of endothelial vasoconstrictors and reduced liberation of vasodilators in the liver contribute to sinusoidal constriction. PH resulting from these factors can be further intensified by a heightened blood flow in the splanchnic circulation. This increased blood flow results from the production of splanchnic vasodilators due to elevated shear stress and diminished effective arterial volume. Consequently, PH is the outcome of both increased resistance to portal venous flow and augmented portal blood flow due to splanchnic vasodilation. As portal pressure remains elevated, collateral vessels develop in an attempt to reduce it [40].

Patients typically remain asymptomatic in the early stages of PH, with hematemesis due to bleeding esophageal varices being the most commonly observed initial presentation. Melena (bloody stools) in the absence of hematemesis may also be present. Given that cirrhosis is a primary cause of PH, patients can exhibit stigmata of cirrhosis, including jaundice, gynecomastia, palmar erythema, spider nevi, testicular atrophy, ascites, pedal edema, or asterixis due to hepatic encephalopathy. Prominent abdominal wall veins may be evident, signifying an effort to redirect portal blood flow through paraumbilical veins into the caval system. In cases of caput medusae, blood flow is directed away from the umbilicus. However, in instances of inferior vena cava obstruction, blood flow is redirected towards the umbilicus to access the superior vena cava system. A venous hum might be detectable in proximity of the xiphoid process or umbilicus. On the other hand, Cruveilhier–Baumgarten syndrome is marked by dilated abdominal wall veins and a faint venous murmur at the umbilicus. Arterial systolic murmurs often result from hepatocellular carcinoma or alcoholic hepatitis. The presence of splenomegaly is a reliable sign in diagnosing PH, and if the spleen is not enlarged, the diagnosis warrants scrutiny. Pancytopenia associated with hypersplenism is caused by reticuloendothelial hyperplasia and does not improve with a portocaval shunt. Although a firm liver suggests cirrhosis, hepatomegaly does not always correlate with the severity of portal hypertension [41].

The evaluation of PH involves gathering a comprehensive patient history and utilizing relevant laboratory data. A complete blood count aids in identifying thrombocytopenia, which results from hypersplenism, as well as anemia caused by gastrointestinal bleeding. A complete metabolic panel aids in identifying renal failure, liver enzyme elevation, which is characteristic of liver disease and viral hepatitis, and hypoalbuminemia. A coagulation profile is used to assess the synthetic function of the liver, with a prolonged prothrombin time and diminished serum albumin level reliably predicting hepatic synthetic function. A Doppler ultrasound of the portal vein can identify stenosis or thrombosis, with results showing either hepatopetal (toward the liver) or hepatofugal (away from the liver) portal vein flow, depending on the degree of PH. Hepatopetal flow is considered normal. An abdominal ultrasound can provide evidence of liver cirrhosis, ascites, and splenomegaly. An endoscopy is mandatory in identifying and, if necessary, treating esophageal or gastric varices. For patients presenting with ascites, paracentesis is performed to determine its cause as well as to exclude spontaneous bacterial peritonitis [42,43].

While measurement of portal pressure is not always necessary for a diagnosis of PH when clinical signs and symptoms are apparent, the openness of the portal and hepatic veins can be evaluated non-invasively through duplex Doppler ultrasound, magnetic resonance, or computed tomography angiography. Direct measurement of portal pressure is invasive, costly, and complex, making indirect methods more preferable. These indirect methods involve balloon occlusion of the hepatic vein and measurement of the wedged hepatic vein pressure, which is then used to calculate the hepatic venous pressure gradient. Other indirect methods of assessing the likelihood of a clinically significant PH are the spleen stiffness, measured by transient elastography [44], as well as the Baveno-VII criteria for PH, in which the platelet count and the liver stiffness (also measured by transient elastography) are taken into account to preview the likelihood of the presence of esophageal varices [45].

### 1.3. Aim of the Review

A growing body of evidence supports the potential profound implications of GM dysbiosis and its associated metabolic products on the pathogenesis and progression of PH [46]. This complex condition, characterized by increased pressure within the portal venous system, poses a multifaceted clinical challenge, manifesting in a spectrum of debilitating complications including variceal bleeding, ascites, hepatic encephalopathy, and hepatorenal syndrome [39]. Given the intricate interplay between various factors contributing to PH, deciphering the underlying mechanisms driving its development is paramount for advancing our understanding of this disorder and devising effective therapeutic strategies to mitigate its devastating impact on patient health and well-being.

In this context, the GLA serves as a pivotal nexus of bidirectional communication, orchestrating a myriad of biochemical and immunological processes that influence both local gut homeostasis and systemic hepatic function [47]. This intricate interplay is underscored by the fact that the liver receives the majority of its blood supply from the portal vein [48], making it uniquely positioned to interface with gut-derived metabolites, microbial products, and immune signals.

Beyond merely unraveling the pathophysiological intricacies of PH, there exists a compelling clinical imperative to enhance diagnostic accuracy, refine prognostic stratification, and optimize therapeutic interventions [49]. PH stands at the convergence of various pathological pathways, where insights derived from dissecting the interplay between the GM and hepatic physiology hold transformative potential for disease management. By elucidating the molecular intricacies of GM–host interactions within the context of PH, an unprecedented opportunity arises to identify novel approaches for early disease detection, elucidate the mechanisms driving disease progression, and pinpoint precisely targeted therapeutic interventions.

In the light of the above, our review endeavors to navigate the labyrinthine network of interactions between the GM, intestinal barrier integrity, and the intricate pathogenesis of PH. Through meticulous examination of the evidence currently in the literature, our aim is to provide a comprehensive overview of current knowledge on the topic, while also identifying gaps that warrant further investigation and discovery. Furthermore, we seek to underscore the clinical relevance of understanding GM-mediated perturbations in the context of PH, emphasizing their potential implications for patient stratification and disease management.

In essence, this review aims to provide a comprehensive synthesis of the current understanding of the complex relationship between the GM and PH via the GLA. For this purpose, here we carefully examine evidence from both human and animal model studies, focusing on investigations that assess GM composition and/or function in the context of PH.

## 2. Materials and Methods

The present review adhered to the PRISMA (Preferred Reporting Items for Systematic Review and Meta-Analysis recommendations) methodology [50]. A systematic literature search was conducted on 21 September 2023, using the PubMed database. The search query employed was as follows: “(gut microbiome OR gut microbiota OR gut-liver axis) AND portal hypertension”. The process led to the assessment of a total of 96 peer-reviewed articles for eligibility. The inclusion criteria were applied within the framework of PICOS (population, intervention, comparison, outcome, study design). The population under investigation encompassed both animal models and human subjects affected by portal hypertension. Interventions ranged from therapeutic modalities targeting gut microbiota to observational assessments of gut microbiota composition and function. Studies comparing the gut microbiota profiles and gut-derived molecules between individuals with portal hypertension and healthy controls, as well as between different stages of portal hypertension, were included. The outcomes of interest included changes in gut microbiota composition, alterations in intestinal barrier function, levels of gut-derived molecules, and progression of portal hypertension and related complications. Overall, we included original research articles employing various study designs such as case-control studies, randomized controlled trials, cross-sectional studies, longitudinal studies, retrospective studies, and animal experiments, while case-reports were excluded. Publications not meeting these criteria were excluded. Moreover, articles were excluded if they were not in English or lacking full-text availability.

To ensure the utmost rigor in the selection process, two authors independently made selections among the papers, and, in instances of disagreement, the ultimate decision was entrusted to a senior author.

**Figure 1:**
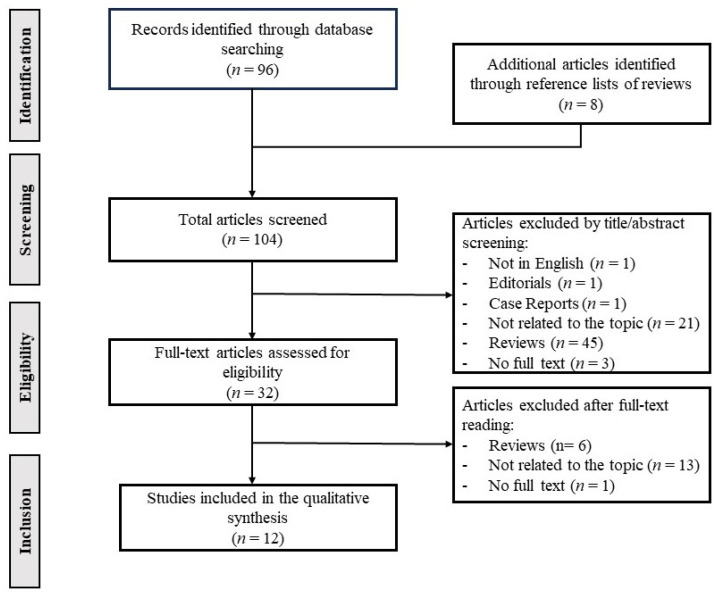
Study selection flowchart.

Consequently, a set of 12 original articles were deemed suitable for inclusion in the qualitative synthesis. Figure 1 provides a visual representation of the study selection process.

## 3. Results

The analyzed papers collectively suggest that there is a relationship between GM, GLA, and PH. This relationship could be orchestrated by several mechanisms such as dysbiosis, bacterial translocation, altered microbial structure, and inflammation. Table 1 reports the principal findings of the six selected papers regarding the animal studies, while Table 2 reports the principal findings of the five selected papers regarding the human studies.

### 3.1. Studies Using Animal Models

In their study, García-Lezana et al. [52] elucidated the remarkable impact of reinstating a healthy intestinal microbiota on the amelioration of PH in a rat model afflicted with MASH. PH was examined with particular focus on its development in the context of MASH, irrespective of liver fibrosis. The transplantation of a healthy microbiota into MASH-afflicted rats yielded substantial reductions in portal pressure, alongside notable enhancements in endothelial function. The investigation further underscored the pivotal role of the farnesoid X receptor axis in regulating the intricate interplay between the gut and liver, thus signifying its potential as a therapeutic target for MASH. The findings from this study propose that the strategic targeting of liver endothelial dysfunction and PH holds significant promise as a therapeutic strategy and a valuable biomarker for monitoring the progression of the disease and gauging treatment efficacy in the context of MASH.

Fecal transplantation experiments, in turn, furnish compelling evidence regarding the pathogenic significance of the GM in the realm of liver maladies. The GM is evidently instrumental in modulating steatosis, inflammation, and fibrosis within various liver disease models. As the authors aptly posited, this study underscores the integral role of the intestinal microbiota in the regulation of PH within a liver disease model. However, it is essential to acknowledge both the merits and demerits of the fecal transplantation approach, with the caveat that its beneficial effects may not endure over the long term.

Moghadamrad et al. [51] presented a study that elucidates the potential influence of the GM on the splanchnic hemodynamic and histological changes associated with PH. Their investigation unveiled the presence of *Lactobacillus murinus* in the spleens of mice afflicted with acute PH, thereby suggesting its potential implication in the pathogenesis of PH. In addition, they endeavored to establish intestinal colonization with the altered Schaedler’s flora (ASF), which comprises a consortium of eight distinct bacterial species. These species were meticulously selected for their prevalence and persistence in the normal microbiota of mice, as well as their amenability to laboratory cultivation [62].

The findings demonstrated that this colonization correlated with an increase in the density of blood vessels within the intestine, a phenomenon that may contribute to the progression of PH. Conversely, the absence of intestinal flora appeared to attenuate the sharp rise in portal pressure following partial portal vein ligation (PPVL). It was also observed that mesenteric artery blood flow increased after PPVL, with no appreciable disparity between the control group and those subjected to ASF colonization. While bacterial translocation has been posited as a crucial mechanism in the pathogenesis of PH, the study did not yield compelling evidence to substantiate significant bacterial translocation. Furthermore, it was noted that intestinal permeability remained largely unaltered following PPVL.

Another study [54] revealed that the oral administration of *Bifidobacterium pseudocatenulatum* CECT7765 to rats with PH resulted in a partial restoration of hemodynamic abnormalities and liver dysfunction. This implies a potential advantageous impact of this specific bifidobacterial strain in mitigating complications associated with cirrhosis.

Paneth cells, situated in the small intestine, play a dual role by secreting substances that combat bacteria and concurrently stimulate blood vessel growth, thereby regulating PH. Indeed, recently, Hassan et al. [56] observed that PH, stemming from heightened resistance to blood flow due to portal vein thrombosis or chronic liver disease, prompted an exploration into the regulatory functions of Paneth cells (PCs) in blood flow and angiogenesis within the context of PH. Depleting Paneth cells led to a reduction in the expression of genes associated with angiogenesis and activation of hypoxia-inducible factors. Intestinal organoids cultured without Paneth cells exhibited diminished blood vessel density. Proteomic analysis elucidated factors released by Paneth cells that induced angiogenic responses in endothelial cells. Furthermore, the depletion of Paneth cells mitigated portal pressure in mice. This study highlighted the interplay between the intestinal microflora and Paneth cell-derived factors in controlling portal pressure and intestinal vascularization. Despite these findings, the precise direct role of Paneth cells in the development of PH remains elusive.

Other data supporting the idea that cirrhosis disrupts the protective barriers that normally prevent bacterial translocation from the intestine to the liver, leading to the entry of bacteria into the portal venous circulation, were provided in animal models by Sorribas et al. [55]. However, the authors revealed that FXR agonists have the potential to mitigate this translocation in cirrhosis. This study emphasized the pivotal role of the gut–liver axis in liver diseases, which is profoundly influenced by bacterial translocation from the gut. In cirrhotic mice, bacterial translocation increases, occurring through both the lymphatic route and the portal venous route. The mucus layer in the ileum serves as a defense mechanism against bacterial translocation; however, cirrhosis disrupts this protective barrier, facilitating pathological bacterial translocation through the intestinal microcirculation and the portal venous route. Notably, FXR agonists demonstrate the capability to reduce bacterial translocation in cirrhosis. In these experiments, some intestine-specific Fxr-null mice were maintained in germ-free or gnotobiotic conditions. Certain outcomes of the research align with previous studies in this field, indeed FXR activation by obethicolic acid (OCA) and fexaramine (Fex) increased the expression of the main tight junction proteins and ileal zonuline, promoting intestinal epithelial cell proliferation and apoptosis. OCA also increased ileal goblet cell numbers, stabilizing epithelial integrity and exerting anti-inflammatory actions. The authors propose that the deficiency in bile acid-induced FXR-signaling may contribute to the observed phenotype in cirrhotic rodents. However, they acknowledge the need for further studies to unravel the paracrine mode of action of bile acids in this process.

The link between the GM and endothelium and PH was also suggested by the paper of Pinheiro et al. [53], reporting that a distinctive bacterial consortium exhibits the potential to ameliorate MASLD, type-2 diabetes, and obesity by reinstating endothelial function, improving insulin signaling, and retarding the progression of these conditions. The authors underscore that metabolic dysregulation is a shared characteristic of MASLD. The study investigated a treatment regimen comprising nine strains of human gut commensal bacteria, demonstrating its efficacy in reducing body weight gain in experimental animals. Notably, MASH, an advancing form of MASLD, currently lacks approved therapeutic interventions. The study postulates that live biotherapeutic products could present a patient-friendly avenue for MASH treatment. Extensive documentation has elucidated the interplay between GM and metabolic syndrome-related disorders, including MASLD. SCFAs, such as butyrate and propionate, produced by gut microbes, have exhibited favorable effects on metabolic diseases. Consequently, the study accentuates the potential of utilizing microbial consortia as a patient-friendly approach for the early-stage treatment of these diseases.

Aller et al. [63] reported that dysbiosis and bacterial translocation observed in an experimental model of liver steatosis induced by pre-hepatic PH in rat imply the presence of a portal hypertensive intestinal microbiome that is potentially involved in both the splanchnic and systemic disturbances associated with pre-hepatic PH. On the other hand, Moghadamrad et al. [51] reported that the lack of gut microbial flora results in unaltered portal pressure but mitigated experimental PH, while bacterial colonization prompts the formation of intestinal mucosal lymphatic and blood vessels, thereby contributing to the onset of PH.

### 3.2. Human Studies

Gitto et al. [59] have observed that transjugular intrahepatic portosystemic shunt (TIPS), which serves as an efficacious intervention for addressing severe complications arising from PH in individuals afflicted with cirrhosis, may also have an effect on GM. Notably, the GM and the bioactive metabolic factors produced by these microorganisms contribute significantly to the etiology of liver diseases and cirrhosis. The amelioration of PH through the placement of TIPS exerts an influence on the composition of GM and the concentration of fecal fatty acids. TIPS implantation leads to a substantial reduction in the portosystemic pressure gradient; however, it does not provoke notable alterations in the levels of SCFAs. The composition of GM can be influenced by cirrhosis and its associated complications. A specific investigation indicated that the installation of TIPS led to an increase in the prevalence of the *Flavonifractor* genus in cirrhotic patients. Nonetheless, further investigation is imperative to elucidate the clinical relevance and significance of this particular bacterial group in the context of cirrhosis. The analysis incorporated a cohort of 13 consecutive patients, encompassing individuals with cirrhosis stemming from various etiologies, all of whom underwent TIPS intervention for refractory cases. While certain findings align with existing knowledge in the field, as the authors suggested, underscoring the pivotal roles played by GM composition and microbial metabolic factors in liver diseases and cirrhosis, it is imperative to acknowledge the limitations of the study. These limitations encompass a relatively small patient population due to stringent selection criteria, with exclusion of individuals experiencing hepatic encephalopathy or receiving antibiotic treatment. Furthermore, it is worth noting that the study’s focus was centered on examining the impact of TIPS on GM within a Western population. Stool samples were collected both before the procedure and 12 weeks post-procedure to capture data under stable conditions.

Recent research has indicated that the administration of proton pump inhibitors (PPIs) to individuals with cirrhosis may heighten the risk of complications, including bacterial infections and hepatic encephalopathy [64]. This occurs through the facilitation of subclinical bacterial translocation from the gastrointestinal tract into the bloodstream. Sturm et al. [64] delved into the exacerbating effects of proton pump inhibitor treatment on bacterial translocation in individuals suffering from advanced cirrhosis and PH. The investigation, encompassing 80 participants with both the conditions, aimed to scrutinize the influence of PPI therapy on subclinical bacterial translocation. The severity of cirrhosis was evaluated using the Model for End-Stage Liver Disease (MELD) and the Freiburg Index of Post-TIPS Survival (FIPS). The study discerned a correlation between PPI treatment and an elevated prevalence of bacterial translocation, underscoring the imperative need for judicious prescribing of PPIs in cirrhosis patients.

They also argued that conventional surrogate markers may not be adept at detecting bacterial translocation in cirrhosis patients and advocated for additional studies to rectify this gap. Notably, patients undergoing PPI treatment exhibited diminished levels of LBP, hinting at a potential increase in Gram-positive bacterial translocation among this cohort.

In human subjects, PH was also studied after liver transplantation. In 2018, Yao et al. [57] investigated the impact of a high portal venous pressure gradient on gut-related bacteremia and subsequent premature death following living-donor liver transplantation. PH can give rise to severe clinical manifestations. A retrospective study discerned that a portal venous pressure gradient higher than 5 mmHg at the conclusion of liver transplantation serves as a predictor for postoperative outcomes, encompassing gut-related bacteremia and early mortality. An elevated portal venous pressure gradient was correlated with an increased incidence of gut-related bacteremia and bacterial translocation. Factors such as pretransplant massive ascites, advanced donor age, and a portal venous pressure gradient exceeding 5 mmHg were predictive of gut-related bacteremia. The monitoring of portal venous pressure gradient during liver transplantation may aid in identifying patients at risk of complications.

However, the authors acknowledged limitations in the study, particularly in the definition of bacterial translocation (BT) and the potential influence of other infections. They emphasized the need for further research to refine the definition of BT and explore the association between biliary infection and PH. The absence of a standardized clinical definition for BT was underscored by the authors, who also noted that gut-related bacteremia might be intertwined with other clinical outcomes like cholangitis. Nearly 39.4% of patients with primary gut-related bacteremia experienced cholangitis. The retrospective nature of the study introduced the possibility of undiagnosed biliary contamination, emphasizing the necessity for additional research to elucidate the mechanism of BT and investigate the connection between biliary infection and PH.

Another human study on subjects with esophago-gastric varices and liver cirrhosis was conducted by Yokoyama et al. [58], who identified differences in the GM between patients with PH and other patients. The study found no significant difference in the GM between healthy controls and patients with colon polyps. However, patients with alcoholic liver cirrhosis had higher levels of *Bifidobacterium*. The researchers aim to further analyze the GM at the species level and develop probiotic treatments.

Patients diagnosed with liver cirrhosis and PH exhibit a distinctive circulating blood microbiome profile characterized by the heightened presence of specific bacterial genera. However, a recent study led by Gedgaudas [60] indicates that this profile does not serve as a reliable predictor for the severity of PH. The research aimed to scrutinize circulating bacterial DNA, inflammatory cytokine levels, and gut permeability markers in individuals with cirrhosis and PH. While certain bacterial genera were present to a greater extent in patients with severe PH, no direct correlation was established between microbiome profiles and the severity of PH. The study also concluded that the circulating microbiome possesses limited potential as a biomarker for predicting PH in cirrhosis patients. Furthermore, the investigation identified alterations in the circulating microbiome among patients with alcoholic hepatitis and hepatitis B-related acute-on-chronic liver failure. Overall, the study underscores the GM’s role in liver diseases and explores the potential utility of circulating bacterial DNA as a noninvasive biomarker. This research included 58 consecutive outpatients diagnosed with stable hepatitis C or alcohol-induced cirrhosis.

The relation with the hepatitis B virus was also investigated by Zhao et al. [61], who reported that gut microbiome and intestinal permeability in cirrhotic patients with chronic hepatitis B virus improve after undergoing a splenectomy plus pericardial devascularization, leading to restoration of the gut microbiome and improved liver function and intestinal permeability.

A further evidence of bacterial translocation occurring during PH was provided by the evidence that the degree of PH has been demonstrated to serve as a prognostic indicator for the occurrence of spontaneous bacterial peritonitis, exhibiting a direct correlation with the levels of bacterial DNA [65,66].

Yokoyama 2020 [58] found that patients with PH had distinct characteristics in their GM compared to healthy controls and non-cirrhosis patients, with significantly higher *Lactobacillales* levels in PH patients. Baffy et al. [46] discussed the potential mechanisms linking GM and PH, highlighting the role of dysbiosis, altered microbial diversity, weakened intestinal barrier, and disrupted host–microbial metabolic interplay, highlighting that it could potentially contribute to an increase in portal pressure from the initial stages of MSLD. Santopaolo et al. [67] emphasized the significance of gut dysbiosis in advanced chronic liver disease with PH and its contribution to complications through inflammatory mechanisms.

## 4. Discussion

PH refers to an anomalous elevation in portal venous pressure, which denotes the blood pressure in the portal vein and its associated branches, responsible for blood drainage from a significant portion of the intestines into the liver. This medical condition is officially defined as the presence of a hepatic venous pressure gradient surpassing 5 mmHg. The primary etiological factor for PH is cirrhosis, a form of chronic liver failure, which accounts for the majority of cases. The majority of clinical complications linked to chronic liver ailments are attributed to PH. PH also leads to increases in mesenteric microvasculature inflammation and vessels’ permeability via several mechanisms including eNOS signaling [68].

**Figure 2:**
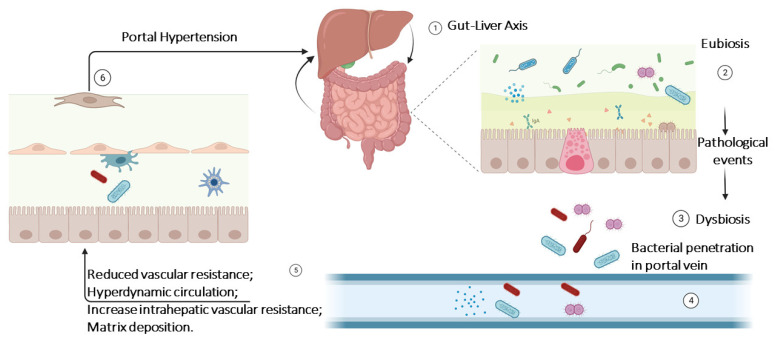
Dysbiosis contribution to portal hypertension. The diagram illustrates the transition from eubiosis, characterized by a balanced gut microbiota, to dysbiosis, wherein microbial equilibriums are disrupted due to various pathological events. Under dysbiotic conditions, immune cell activation and the production of metabolites by both host and microbiome species lead to increased gut permeability, facilitating bacterial translocation into the portal veins. Consequently, this cascade of events triggers processes that diminish vascular resistance, enhance circulation dynamics, elevate intrahepatic vascular resistance, and ultimately result in increased matrix deposition in hepatic vessels, culminating in the onset of portal hypertension. This pathological state disrupts the normal gut–liver axis, exacerbating its self-propagating effects.

The majority of the analyzed papers regarding GM, GLA, and PH are related to cirrhosis-related PH (Figure 2).

Cirrhosis is linked to a significant dysfunction of the gut barrier, mirroring disease progression [47]. In cases of compensated cirrhosis, the manifestations of barrier dysfunction are hardly distinguishable from those observed in various etiologies of chronic liver disease. In stark contrast, the disruption of the gut barrier in decompensated cirrhosis stems from damage at all levels of the intestinal defense system, irrespective of etiology, and is linked to liver insufficiency, diminished bile flow, and compromised immune functions [47]. Both the GM and barrier dysfunction play direct roles in the pathogenesis of compensated cirrhosis. In decompensated cirrhosis, however, both factors are intricately connected with the occurrence and severity of complications, particularly bacterial infections and encephalopathy [47].

For decades, deviations in GM have been acknowledged in individuals and experimental models with cirrhosis [69]. In recent times, metagenomic methodologies have profiled the fecal microbiome in cirrhosis as one characterized by lower diversity, a heightened relative prevalence of potentially pathogenic taxa (e.g., *Enterococcaceae, Staphylococcaceae*, and notably *Enterobacteriaceae*), and a diminished abundance of potentially beneficial autochthonous taxa (such as *Lachnospiraceae* and *Ruminococcaceae*) [70,71]. This altered microbiome structure in cirrhosis appears increasingly pronounced as the disease progresses, intensifying with decompensation and correlating with unfavorable outcomes [72]. Disruptions in the GM lay the groundwork for gut barrier dysfunction in cirrhosis. Shifts in GM composition in cirrhosis result from the disturbance of multiple factors governing the microbiome, including the decreased motility and transit time of the small bowel, particularly in the ascitic stage, as a primary contributor to dysbiosis [73,74,75]; the irregularities in bile acid levels, encompassing reduced primary bile acids and elevated secondary bile acids in the gut [76,77,78]; and compromised intestinal immune responses. Experimental cirrhosis accompanied by ascites is characterized by an imbalance in Paneth cell α-defensins and hindered dendritic cell function, with a notably exacerbated severity in rats with ascites and pathological bacterial translocation. Hypochlorhydria in cirrhosis, even without proton pump inhibition, is an additional factor contributing to microbiota alterations [79,80,81]. Notably, the configuration of microbiota abnormalities in cirrhosis remains consistent across various etiologies [82]. A distinctive hallmark of cirrhosis is the migration of bacteria from the oral cavity into the intestine. An enrichment of patient stools with species originating from the mouth and *Lactobacillaceae* appears to be connected to shifts in salivary microbiota, the use of proton pump inhibitors, and relatively low gastric acid levels. Moreover, an increase in *Lactobacillaceae* has been described in studies of the GM in cirrhosis, which may be associated with lactulose use [83].

Cirrhosis is also linked to harm to the physical and immunological elements of the intestinal barrier. Augmented permeability throughout the gastroduodenal, small intestine, colon, and the entire intestine, as an indication of gut barrier disruption, are well-documented characteristics of cirrhosis, particularly in the presence of ascites [84]. This impairment of the gut barrier leads to an enhanced passage of macromolecules, including bacterial elements such as lipopolysaccharides (LPS) or bacterial DNA, along with viable bacteria (referred to as bacterial translocation), into the systemic circulation [85]. The damage to the intestinal barrier progresses in parallel with the advancement of cirrhosis and is especially severe when ascites and gut bacterial translocation are present [86,87]. There is ongoing debate regarding the mechanism through which gut bacteria gain access to the internal environment in cirrhosis. It is well established that pathological movement of live bacteria from the intestinal lumen into the mesenteric lymph nodes as well as into the systemic circulation occurs [88,89]. Recent evidence suggests that in cirrhosis, this lymphatic way of translocation exists together with the portal venous translocation of bacteria and bacterial products to the liver due to a disruption of the gut–vascular barrier [55]. The increased vascular permeability occurs regardless of lymphatic pathways and pH levels, as it is solely evident in models featuring liver dysfunction.

Interestingly, obeticholic acid has been shown to recover diminished FXR signaling in the ileum, enhance the mucus functionality, and stabilize the gut–vascular barrier in cirrhotic rats, supporting the idea that the nuclear receptor FXR plays a partial role in modulating mucus and the gut–vascular barrier in cirrhosis [55]. Furthermore, obeticholic acid and other FXR agonists restore microbiota composition, enhance intestinal innate defenses, mitigate intestinal inflammation, and lower bacterial translocation and endotoxemia in experimental cirrhosis [55,90,91]. Reduced ileal FXR signaling is a probable result of a decrease in primary bile acids and a rise in secondary bile acids in the gut, in addition to intestinal inflammation [55,92,93]. The aforementioned irregularities in intestinal barrier function in cirrhosis have been associated with alterations in the intestinal structure, encompassing submucosal edema, modest immune cell infiltration, and the disorganization of interepithelial tight junction proteins in humans and experimental models of cirrhosis [86,87,93,94,95,96]. According to current research, subclinical intestinal inflammation resulting from changes in GM exacerbates barrier dysfunction in advanced cirrhosis. While cirrhosis evolves into an ascitic stage, the intestinal immune system in cirrhotic rats undergoes a shift toward a Th1 regulatory pattern, marked by the expansion of TNF-α- and IFN-γ-expressing lymphocytes, and the concomitant depletion of Th17 cells in the lamina propria [86]. Intestinal decontamination shifts the balance of GM composition, alleviates the pro-inflammatory response of mucosal immune cells, and lessens intestinal permeability and bacterial translocation, underscoring the pivotal role of microbiota alterations in intestinal inflammation in cirrhosis.

Moreover, preliminary preclinical evidence utilizing experimental manipulation of the GM through fecal microbiome transplantation has revealed the autonomous involvement of the GM in the progression of diet-induced hepatic steatosis [97] as well as the modulation of fibrosis [98].

Currently, the exact link between GM and PH is hard to define. However, the link between GM, GLA, and cirrhosis is well founded and documented, and cirrhosis is the major cause of PH, but the certainty of the association between the reported findings and the presence of PH is not as robust because of the frequent underlying cirrhosis condition. In fact, very few papers, and almost all of them on animal models, have probed GM changes in PH from pre- or post-hepatic causes. The present results in the literature, thus, suffer from a co-occurrence bias between cirrhosis and PH that does not allow disambiguation of this association. This aspect represents a major limitation of the work in the literature that needs further investigation in the near future.

## 5. Conclusions

In recent years, our comprehension of the GM’s role in human ailments has undergone substantial advancement. The increasing accessibility of intricate methodologies involving integrated metagenomics and metabolomics analyses has afforded a more comprehensive perspective, thereby facilitating the discovery of novel molecular targets capable of altering the narrative of diseases, shifting the focus from diagnosis to treatment. Metabolomics has illuminated the significance of functional diversity within gut microbiome enzymatic activities, diverging from a standpoint predominantly reliant on compositional analysis. Nonetheless, a substantial knowledge gap persists regarding the influence of the GLA on the onset and progression of PH. Mounting evidence suggests that perturbations within the GLA, particularly arising from dysbiosis, damage to the intestinal barrier leading to heightened permeability, and modifications in the enterohepatic circulation of bile acids, underlie the genesis of PH. Analyzing microbial constituents traversing the intestinal barrier may expediently enable patient stratification based on systemic inflammatory and hemodynamic conditions. Addressing the unmet need of identifying the GLA-related metabolic and molecular pathways underpinning this process holds potential not only for elucidating pathogenesis and prognostication but also for directing novel therapeutic interventions. Modulating the intestinal milieu through FMT emerges as a highly promising tool in PH treatment, as evidenced by compelling outcomes in animal models. Nevertheless, the necessity remains for randomized controlled trials in human subjects to substantiate its efficacy and clarify its mechanisms of action. Finally, exploring the interplay between the GM and various available pharmacological therapies stands as a valuable approach for monitoring treatment effectiveness as a noninvasive predictor of hemodynamic response, thereby transitioning towards a personalized therapeutic paradigm, with profound implications for patient prognosis and survival.

## Figures and Tables

**Table 1:** Animal studies. Abbreviations: eNOS: endothelial nitric oxide synthase; FEX: fexaramine; FITC-dextran: fluorescein isothiocyanate-dextran; FXR: farnesoid X receptor; GFP-*E. coli*: green fluorescent protein-*E. coli*; GM: gut microbiota; LBP: live biotherapeutic products; NALFD: non-alcoholic fatty liver disease; NASH: non-alcoholic steatohepatitis; OCA: obethicolic acid; PH: portal hypertension.

Paper Title	Measured Outcomes	Main Findings	Limitations	Country	Animal Model
Attenuated Portal Hypertension in Germ-Free Mice: Function of Bacterial Flora on the Development of Mesenteric Lymphatic and Blood Vessels [51]	Portal pressure and portosystemic shunts.	Experimental pre-hepatic PH is significantly attenuated in the complete absence of gut microbial flora. Presence of intestinal mucosal lymphatic and blood vessels induced by bacterial colonization may contribute to the development of PH.	The limitations of the study are not explicitly stated, but the paper suggests that the short colonization time may have affected the results and that further investigation into the contribution of Paneth cells to the development of intestinal lymphatic vessels is needed.	Switzerland	Germ-free male C57BL/6 mice, ASF mice, and SPF mice, aged 10–12 weeks
Restoration of a Healthy Intestinal Microbiota Normalizes Portal Hypertension in a Rat Model of Nonalcoholic Steatohepatitis [52]	Development and progression of PH in the specific context of NASH, as well as the influence of the GM on PH, insulin resistance, and endothelial dysfunction.	The GM has a direct influence on the development of PH in rats with diet-induced NASH and dysbiosis; PH, insulin resistance, and endothelial dysfunction revert when a healthy GM is restored.	The limitations of the study include: - The need to focus on changes in fibrosis as the main endpoint in clinical trials hinders the development of effective drug therapies for NASH. - The lack of specific biomarkers for NASH that appear early in the disease, respond sensitively to therapies, and correlate with clinical outcomes is a major challenge. - The beneficial effects of GM transplantation are unlikely to extend in time, as the eubiotic GM would likely drift back to dysbiosis under the same diet that induced the model phenotype. Therefore, the value of GM transplantation should be considered as proof-of-concept rather than a potential long-term therapeutic option.	Spain	Male Sprague Dawley rats
A Nine-Strain Bacterial Consortium Improves Portal Hypertension and Insulin Signaling and Delays NAFLD Progression In Vivo [53]	Improvement in PH and endothelial function in NASH rats, improvement in liver fibrosis in the STAM™ mouse model, and a decrease in steatosis, ballooning, and serum cytokeratin-18 levels.	The main findings are that the defined bacterial consortium improved PH and endothelial function in NASH rats, modulated pro-fibrogenic pathways, and improved liver fibrosis in the STAM™ mouse model.	The limitations of the study include: - Lack of improvement in NASH histopathology despite observed improvements in symptomatology and prevention of progression to fibrosis - Need for further work to confirm the mode-of-action behind the consortium treatment - Lack of approved therapies for NASH, indicating the challenging nature of the condition - Disputed role of the gut microbiome in NAFLD and other metabolic diseases, suggesting ongoing research is needed - The study convincingly showed that live LBP are a patient-friendly approach worth considering for development as therapy for NASH, indicating a potential bias towards the effectiveness of LBP	Spain/Japan	Male Sprague Dawley OFA rats and C57 BL/6J male mice
Improved hemodynamic and liver function in portal hypertensive cirrhotic rats after administration of *B. pseudocatenulatum* CECT 7765 [54]	Beneficial effect of *B. pseudocatenulatum* CECT7765 in reducing complications derived from PH in cirrhosis, including reduction of the Firmicutes/Bacteroidetes ratio, improvement in hemodynamic parameters, improvement in liver function markers, reduction in systemic inflammatory mediators, and increased gene expression levels of FXR and eNOS in the liver.	- The bifidobacterial strain *B. pseudocatenulatum* CECT7765 showed a beneficial effect in reducing complications derived from PH in cirrhosis. - Oral intervention with *B. pseudocatenulatum* CECT7765 in bile-duct-ligated rats improved hemodynamic and liver function parameters associated with the modification of the GM content. - The observed effect of the bifidobacterial strain on liver FXR and eNOS expression suggests a possible mechanism for inducing beneficial effects, which requires further studies.	The limitations of the study include the fact that it was conducted on rats, the need for further studies to evaluate the effects of the bifidobacterial strain on humans, the lack of information on long-term effects and potential adverse effects, and the absence of discussion on potential interactions with other medications or individual variations in response. Additionally, the study does not provide information on the optimal dosage or duration of treatment with the bifidobacterial strain.	Spain	Male Sprague Dawley rats
FXR modulates the gut-vascular barrier by regulating the entry sites for bacterial translocation in experimental cirrhosis [55]	Cirrhosis impairs barriers; FXR agonists reduce bacterial translocation; microbiome plays a key role in mucus function and barrier; study aimed to characterize changes in barriers and FXR’s role (confidence: 90).	OCA significantly reduced pathological translocation of GFP-*E. coli* from the ileal lumen to the liver in cirrhotic mice. Fex also significantly reduced pathological translocation of GFP-*E. coli* from the ileal lumen to the liver in cirrhotic mice. FxrΔIEC mice did not show any translocation of even small 4 kDa FITC-dextran or GFP-*E. coli* to the liver. Performing PPVL on FxrΔIEC mice led to a small but evident increase in intrahepatically recoverable 4 kDa FITC-dextran. In individual PPVL- FxrΔIEC-mice, GFP-*E. coli* was retrievable intrahepatically.	Not reported	Switzerland	Mice C57BL/6J (female)
Paneth cells promote angiogenesis and regulate portal hypertension in response to microbial signals [56]	Paneth cells play a critical role in preventing dysbiosis and regulating PH and angiogenesis.	Effect of PC depletion on portal pressure, intestinal and mesenteric angiogenesis in vivo, as well as its in vitro angiogenic effects in response to microbial stimuli.	Not reported	Switzerland	Mice; Math1Lox/Lox VilCreERT2

**Table 2:** Human studies. Abbreviations: ALDLT: adult-to-adult living donor liver transplantation; GM: gut microbiota; MCFA: medium-chain fatty acid; NALFD: non-alcoholic fatty liver disease; NASH: non-alcoholic steatohepatitis; PH: portal hypertension; PPI: proton pump inhibitor; PPVL: pre-hepatic portal-hypertensive; PVPG: portal venous pressure gradient; SCFA: short-chain fatty acid; TIPS: transjugular intrahepatic portosystemic shunt.

Paper Title	Measured Outcomes	Main Findings	Limitations	Country	Study Design	Population
A High Portal Venous Pressure Gradient Increases Gut-Related Bacteremia and Consequent Early Mortality After Living Donor Liver [57]	The main or primary outcomes measured in the study are the incidence of bacteremia, 90-day mortality rate, and 1-year survival.	1. A high PVPG (>5 mmHg) at the end of ALDLT is significantly associated with a higher incidence of bacteremia, increased 90-day mortality rate, and poorer 1-year survival. 2. A PVPG greater than 5 mmHg is an independent predictor of bacteremia due to gut bacteria. 3. Monitoring the PVPG is clinically meaningful for predicting patients’ prognosis, particularly in relation to the occurrence of gut-related bacteremia and early mortality.	The limitations of the study include an imperfect definition of bacterial translocation, potential associations between gut-related bacteremia and other clinical outcomes not explored in the study, and the need for further investigation into the association between biliary infection and PH.	Japan	Retrospective	223 adults underwent primary living donor transplantation (110 female, 113 male)
Exploratory Research on the Relationship between Human Gut Microbiota and Portal Hypertension [58]	Characteristics of the GM in PH patients with esophago-gastric varices and liver cirrhosis.	The main findings of the study are the significant differences in GM composition between patients with PH and other patients, including higher levels of *Lactobacillales* and lower levels of *Clostridium cluster IV*, as well as the unexpected lack of decrease in the *Bifidobacterium* genus in patients with PH. The study also highlighted an increase in *Streptococcus* and a decrease in SCFAs in patients with PH.	- Single-center study. - Small sample size. - Cross-sectional study. - Lack of analysis of *Helicobacter pylori* infection rate. - Difficulty in distinguishing between PH and liver cirrhosis due to their relationship in all patients. - Suggestion for further research using methods that evaluate bacteria at the species level (e.g., intestinal flora analysis method and next-generation sequencing).	Japan	Exploratory	36 patients (12 patients with portal hypertension, 12 healthy controls, and 12 non-cirrhosis patients) (22 male, 14 female)
Transjugular Intrahepatic Porto-Systemic Shunt Positively Influences the Composition and Metabolic Functions of the Gut Microbiota in Cirrhotic Patients [59]	The main or primary outcome measured in the study is the modification of GM composition and fecal concentration of pro-inflammatory MCFAs following correction of PH through TIPS placement.	1. TIPS placement resulted in a significant reduction in portal–caval pressure gradient. 2. Following TIPS, there were increased levels of beneficial *Flavonifractor* spp. and decreased levels of *Clostridiaceae*, which are linked to abdominal infections in cirrhotic patients. 3. There were decreased levels of pro-inflammatory MCFAs after TIPS.	The limitations of the study include a small sample size, potential influence of alcohol-use disorder on GM despite abstinence, limited generalizability to populations with different diets and ethnicities, and the novelty of the fecal fatty acid analysis not previously conducted in similar studies.	Italy	Prospective	23 cirrhotic patients receiving TIPS (11 male, 12 female)
Circulating microbiome in patients with portal hypertension [60]	This study examined the presence of bacterial DNA patterns, inflammatory cytokines, and indicators of gut permeability in both peripheral and hepatic veins, among individuals diagnosed with cirrhosis and PH.	- The circulating plasma microbiome profiles in patients with cirrhosis were distinct from those of the controls, characterized by specific bacterial genera enrichment and depletion. Enrichment of certain bacterial genera was associated with severe PH, but the circulating microbiome profiles could not predict the severity of PH.	The limitations of the study include: - Lack of paired fecal samples for correlation with gut and circulating blood microbiome data. - Potential bias due to PPI treatment and alcohol consumption in the patient cohort. - Cross-sectional study design with samples collected at a single time point. - Potential contamination of low-biomass samples at various stages of sample processing. - Inability of circulating microbial composition to predict the severity of PH.	Lithuania	Case-control	58 patients with liver cirrhosis (23 female, 35 male) and 46 control (36 female; 10 male)
Improvement of gut microbiome and intestinal permeability following splenectomy plus pericardial devascularization in hepatitis B virus-related cirrhotic portal hypertension [61]	Intestinal permeability and systemic inflammatory levels measured by DAO, D-LA, LPS, and TNF-αconcentrations.	- The study evaluated the gut microbiome and intestinal permeability status in HBV-related cirrhotic patients after undergoing an SPD, showing significant differences from healthy controls. - The study demonstrated that gut microbiome dysbiosis, increased intestinal permeability, and impaired liver function were significantly mitigated at 12 months after surgery, likely related to restoring the gut microbiome. - The findings suggest that understanding the risks and beneficial effects of SPD for cirrhotic patients from the perspective of their intestinal microenvironments is crucial.	The limitations of the study include the focus on HBV-related cirrhotic patients, exclusion of patients with different etiologies, potential influence of individual differences and unpredictable factors, and the use of 16s rRNA gene sequencing instead of metagenomic sequencing.	China	Case-control	34 HBV-related cirrhotic patients and 20 healthy controls

## Data Availability

No new data were created or analyzed in this study. Data sharing is not applicable to this article.

## References

[1] Bidell M.R., Hobbs A.L.V., Lodise T.P. (2022). Gut Microbiome Health and Dysbiosis: A Clinical Primer. Pharmacotherapy.

[2] O’Hara A.M., Shanahan F. (2006). The Gut Flora as a Forgotten Organ. EMBO Rep..

[3] Jandhyala S.M. (2015). Role of the Normal Gut Microbiota. World J. Gastroenterol..

[4] Perler B.K., Friedman E.S., Wu G.D. (2023). The Role of the Gut Microbiota in the Relationship Between Diet and Human Health. Annu. Rev. Physiol..

[5] Carding S., Verbeke K., Vipond D.T., Corfe B.M., Owen L.J. (2015). Dysbiosis of the Gut Microbiota in Disease. Microb. Ecol. Health Dis..

[6] Tarao K., So K., Moroi T., Ikeuchi T., Suyama T., Endo O., Fukushima K. (1977). Detection of Endotoxin in Plasma and Ascitic Fluid of Patients with Cirrhosis: Its Clinical Significance. Gastroenterology.

[7] Triger D.R., Boyer T.D., Levin J. (1978). Portal and Systemic Bacteraemia and Endotoxaemia in Liver Disease. Gut.

[8] Chen B., Chen H., Shu X., Yin Y., Li J., Qin J., Chen L., Peng K., Xu F., Gu W. (2018). Presence of Segmented Filamentous Bacteria in Human Children and Its Potential Role in the Modulation of Human Gut Immunity. Front. Microbiol..

[9] Blacher E., Levy M., Tatirovsky E., Elinav E. (2017). Microbiome-Modulated Metabolites at the Interface of Host Immunity. J. Immunol..

[10] Levy M., Blacher E., Elinav E. (2017). Microbiome, Metabolites and Host Immunity. Curr. Opin. Microbiol..

[11] Tsilingiri K., Rescigno M. (2013). Postbiotics: What Else?. Benef. Microbes.

[12] Johansson M.E.V. (2012). Fast Renewal of the Distal Colonic Mucus Layers by the Surface Goblet Cells as Measured by in Vivo Labeling of Mucin Glycoproteins. PLoS ONE.

[13] Gibbins H.L., Proctor G.B., Yakubov G.E., Wilson S., Carpenter G.H. (2015). SIgA Binding to Mucosal Surfaces Is Mediated by Mucin-Mucin Interactions. PLoS ONE.

[14] Vaishnava S., Yamamoto M., Severson K.M., Ruhn K.A., Yu X., Koren O., Ley R., Wakeland E.K., Hooper L.V. (2011). The Antibacterial Lectin RegIIIγ Promotes the Spatial Segregation of Microbiota and Host in the Intestine. Science.

[15] Bergström J.H., Birchenough G.M.H., Katona G., Schroeder B.O., Schütte A., Ermund A., Johansson M.E.V., Hansson G.C. (2016). Gram-Positive Bacteria Are Held at a Distance in the Colon Mucus by the Lectin-like Protein ZG16. Proc. Natl. Acad. Sci. USA.

[16] Jakobsson H.E., Rodríguez-Piñeiro A.M., Schütte A., Ermund A., Boysen P., Bemark M., Sommer F., Bäckhed F., Hansson G.C., Johansson M.E.V. (2015). The Composition of the Gut Microbiota Shapes the Colon Mucus Barrier. EMBO Rep..

[17] Derrien M., Van Baarlen P., Hooiveld G., Norin E., Müller M., de Vos W.M. (2011). Modulation of Mucosal Immune Response, Tolerance, and Proliferation in Mice Colonized by the Mucin-Degrader *Akkermansia muciniphila*. Front. Microbiol..

[18] Desai M.S., Seekatz A.M., Koropatkin N.M., Kamada N., Hickey C.A., Wolter M., Pudlo N.A., Kitamoto S., Terrapon N., Muller A. (2016). A Dietary Fiber-Deprived Gut Microbiota Degrades the Colonic Mucus Barrier and Enhances Pathogen Susceptibility. Cell.

[19] Kurashima Y., Kiyono H. (2017). Mucosal Ecological Network of Epithelium and Immune Cells for Gut Homeostasis and Tissue Healing. Annu. Rev. Immunol..

[20] Spadoni I., Fornasa G., Rescigno M. (2017). Organ-Specific Protection Mediated by Cooperation between Vascular and Epithelial Barriers. Nat. Rev. Immunol..

[21] Spadoni I., Zagato E., Bertocchi A., Paolinelli R., Hot E., Di Sabatino A., Caprioli F., Bottiglieri L., Oldani A., Viale G. (2015). A Gut-Vascular Barrier Controls the Systemic Dissemination of Bacteria. Science.

[22] Mouries J., Brescia P., Silvestri A., Spadoni I., Sorribas M., Wiest R., Mileti E., Galbiati M., Invernizzi P., Adorini L. (2019). Microbiota-Driven Gut Vascular Barrier Disruption Is a Prerequisite for Non-Alcoholic Steatohepatitis Development. J. Hepatol..

[23] Rivera C.A., Adegboyega P., van Rooijen N., Tagalicud A., Allman M., Wallace M. (2007). Toll-like Receptor-4 Signaling and Kupffer Cells Play Pivotal Roles in the Pathogenesis of Non-Alcoholic Steatohepatitis. J. Hepatol..

[24] Saberi M., Woods N.-B., de Luca C., Schenk S., Lu J.C., Bandyopadhyay G., Verma I.M., Olefsky J.M. (2009). Hematopoietic Cell-Specific Deletion of Toll-like Receptor 4 Ameliorates Hepatic and Adipose Tissue Insulin Resistance in High-Fat-Fed Mice. Cell Metab..

[25] Miura K., Kodama Y., Inokuchi S., Schnabl B., Aoyama T., Ohnishi H., Olefsky J.M., Brenner D.A., Seki E. (2010). Toll-Like Receptor 9 Promotes Steatohepatitis by Induction of Interleukin-1β in Mice. Gastroenterology.

[26] Henao-Mejia J., Elinav E., Jin C., Hao L., Mehal W.Z., Strowig T., Thaiss C.A., Kau A.L., Eisenbarth S.C., Jurczak M.J. (2012). Inflammasome-Mediated Dysbiosis Regulates Progression of NAFLD and Obesity. Nature.

[27] Dumas M.-E., Barton R.H., Toye A., Cloarec O., Blancher C., Rothwell A., Fearnside J., Tatoud R., Blanc V., Lindon J.C. (2006). Metabolic Profiling Reveals a Contribution of Gut Microbiota to Fatty Liver Phenotype in Insulin-Resistant Mice. Proc. Natl. Acad. Sci. USA.

[28] Chen Y.-M., Liu Y., Zhou R.-F., Chen X.-L., Wang C., Tan X.-Y., Wang L.-J., Zheng R.-D., Zhang H.-W., Ling W.-H. (2016). Associations of Gut-Flora-Dependent Metabolite Trimethylamine-N-Oxide, Betaine and Choline with Non-Alcoholic Fatty Liver Disease in Adults. Sci. Rep..

[29] Zhu L., Baker S.S., Gill C., Liu W., Alkhouri R., Baker R.D., Gill S.R. (2013). Characterization of Gut Microbiomes in Nonalcoholic Steatohepatitis (NASH) Patients: A Connection between Endogenous Alcohol and NASH. Hepatology.

[30] Engstler A.J., Aumiller T., Degen C., Dürr M., Weiss E., Maier I.B., Schattenberg J.M., Jin C.J., Sellmann C., Bergheim I. (2016). Insulin Resistance Alters Hepatic Ethanol Metabolism: Studies in Mice and Children with Non-Alcoholic Fatty Liver Disease. Gut.

[31] Hoyles L., Fernández-Real J.-M., Federici M., Serino M., Abbott J., Charpentier J., Heymes C., Luque J.L., Anthony E., Barton R.H. (2018). Molecular Phenomics and Metagenomics of Hepatic Steatosis in Non-Diabetic Obese Women. Nat. Med..

[32] Koh A., De Vadder F., Kovatcheva-Datchary P., Bäckhed F. (2016). From Dietary Fiber to Host Physiology: Short-Chain Fatty Acids as Key Bacterial Metabolites. Cell.

[33] Sawicki C.M., Livingston K.A., Obin M., Roberts S.B., Chung M., McKeown N.M. (2017). Dietary Fiber and the Human Gut Microbiota: Application of Evidence Mapping Methodology. Nutrients.

[34] Wang J., Qin J., Li Y., Cai Z., Li S., Zhu J., Zhang F., Liang S., Zhang W., Guan Y. (2012). A Metagenome-Wide Association Study of Gut Microbiota in Type 2 Diabetes. Nature.

[35] Zhao L., Zhang F., Ding X., Wu G., Lam Y.Y., Wang X., Fu H., Xue X., Lu C., Ma J. (2018). Gut Bacteria Selectively Promoted by Dietary Fibers Alleviate Type 2 Diabetes. Science.

[36] Jiao N., Baker S.S., Chapa-Rodriguez A., Liu W., Nugent C.A., Tsompana M., Mastrandrea L., Buck M.J., Baker R.D., Genco R.J. (2018). Suppressed Hepatic Bile Acid Signalling despite Elevated Production of Primary and Secondary Bile Acids in NAFLD. Gut.

[37] Ferslew B.C., Xie G., Johnston C.K., Su M., Stewart P.W., Jia W., Brouwer K.L.R., Sidney Barritt A. (2015). Altered Bile Acid Metabolome in Patients with Nonalcoholic Steatohepatitis. Dig. Dis. Sci..

[38] Gilbert A., Carnot P. (1902). Les Fonctions Hépatiques.

[39] Berzigotti A., Seijo S., Reverter E., Bosch J. (2013). Assessing Portal Hypertension in Liver Diseases. Expert Rev. Gastroenterol. Hepatol..

[40] García-Pagán J.-C., Gracia-Sancho J., Bosch J. (2012). Functional Aspects on the Pathophysiology of Portal Hypertension in Cirrhosis. J. Hepatol..

[41] Schuppan D., Afdhal N.H. (2008). Liver Cirrhosis. Lancet.

[42] Diaz K.E., Schiano T.D. (2019). Evaluation and Management of Cirrhotic Patients Undergoing Elective Surgery. Curr. Gastroenterol. Rep..

[43] Alukal J.J., Thuluvath P.J. (2019). Gastrointestinal Failure in Critically Ill Patients with Cirrhosis. Am. J. Gastroenterol..

[44] Dajti E., Ravaioli F., Zykus R., Rautou P.-E., Elkrief L., Grgurevic I., Stefanescu H., Hirooka M., Fraquelli M., Rosselli M. (2023). Accuracy of Spleen Stiffness Measurement for the Diagnosis of Clinically Significant Portal Hypertension in Patients with Compensated Advanced Chronic Liver Disease: A Systematic Review and Individual Patient Data Meta-Analysis. Lancet Gastroenterol. Hepatol..

[45] De Franchis R., Bosch J., Garcia-Tsao G., Reiberger T., Ripoll C., Abraldes J.G., Albillos A., Baiges A., Bajaj J., Bañares R. (2022). Baveno VII–Renewing Consensus in Portal Hypertension. J. Hepatol..

[46] Baffy G. (2019). Potential Mechanisms Linking Gut Microbiota and Portal Hypertension. Liver Int..

[47] Albillos A., De Gottardi A., Rescigno M. (2020). The Gut-Liver Axis in Liver Disease: Pathophysiological Basis for Therapy. J. Hepatol..

[48] Carneiro C., Brito J., Bilreiro C., Barros M., Bahia C., Santiago I., Caseiro-Alves F. (2019). All about Portal Vein: A Pictorial Display to Anatomy, Variants and Physiopathology. Insights Imaging.

[49] Simonetto D.A., Liu M., Kamath P.S. (2019). Portal Hypertension and Related Complications: Diagnosis and Management. Mayo Clin. Proc..

[50] Moher D., Liberati A., Tetzlaff J., Altman D.G. (2009). The PRISMA Group Preferred Reporting Items for Systematic Reviews and Meta-Analyses: The PRISMA Statement. PLoS Med..

[51] Moghadamrad S., McCoy K.D., Geuking M.B., Sägesser H., Kirundi J., Macpherson A.J., De Gottardi A. (2015). Attenuated Portal Hypertension in Germ-free Mice: Function of Bacterial Flora on the Development of Mesenteric Lymphatic and Blood Vessels. Hepatology.

[52] García-Lezana T., Raurell I., Bravo M., Torres-Arauz M., Salcedo M.T., Santiago A., Schoenenberger A., Manichanh C., Genescà J., Martell M. (2018). Restoration of a Healthy Intestinal Microbiota Normalizes Portal Hypertension in a Rat Model of Nonalcoholic Steatohepatitis. Hepatology.

[53] Pinheiro I., Barberá A., Raurell I., Estrella F., De Leeuw M., Bolca S., Gottardi D., Horscroft N., Possemiers S., Salcedo M.T. (2022). A Nine-Strain Bacterial Consortium Improves Portal Hypertension and Insulin Signaling and Delays NAFLD Progression In Vivo. Biomedicines.

[54] Gómez-Hurtado I., Zapater P., Portune K., Juanola O., Fernández-Iglesias A., González-Navajas J.M., Gracia-Sancho J., Sanz Y., Francés R. (2019). Improved Hemodynamic and Liver Function in Portal Hypertensive Cirrhotic Rats after Administration of *B. pseudocatenulatum* CECT 7765. Eur. J. Nutr..

[55] Sorribas M., Jakob M.O., Yilmaz B., Li H., Stutz D., Noser Y., de Gottardi A., Moghadamrad S., Hassan M., Albillos A. (2019). FXR Modulates the Gut-Vascular Barrier by Regulating the Entry Sites for Bacterial Translocation in Experimental Cirrhosis. J. Hepatol..

[56] Hassan M., Moghadamrad S., Sorribas M., Muntet S.G., Kellmann P., Trentesaux C., Fraudeau M., Nanni P., Wolski W., Keller I. (2020). Paneth Cells Promote Angiogenesis and Regulate Portal Hypertension in Response to Microbial Signals. J. Hepatol..

[57] Yao S., Yagi S., Uozumi R., Iida T., Nagao M., Okamura Y., Anazawa T., Okajima H., Kaido T., Uemoto S. (2018). A High Portal Venous Pressure Gradient Increases Gut-Related Bacteremia and Consequent Early Mortality After Living Donor Liver Transplantation. Transplantation.

[58] Yokoyama K., Tsuchiya N., Yamauchi R., Miyayama T., Uchida Y., Shibata K., Fukuda H., Umeda K., Takata K., Tanaka T. (2020). Exploratory Research on the Relationship between Human Gut Microbiota and Portal Hypertension. Intern. Med..

[59] Gitto S., Vizzutti F., Baldi S., Campani C., Navari N., Falcini M., Venturi G., Montanari S., Roccarina D., Arena U. (2023). Transjugular Intrahepatic Porto-Systemic Shunt Positively Influences the Composition and Metabolic Functions of the Gut Microbiota in Cirrhotic Patients. Dig. Liver Dis..

[60] Gedgaudas R., Bajaj J.S., Skieceviciene J., Varkalaite G., Jurkeviciute G., Gelman S., Valantiene I., Zykus R., Pranculis A., Bang C. (2022). Circulating Microbiome in Patients with Portal Hypertension. Gut Microbes.

[61] Zhao Y., Zhou R., Guo Y., Chen X., Zhang A., Wang J., Ji F., Qin B., Geng J., Kong G. (2022). Improvement of Gut Microbiome and Intestinal Permeability Following Splenectomy plus Pericardial Devascularization in Hepatitis B Virus-Related Cirrhotic Portal Hypertension. Front. Immunol..

[62] Dewhirst F.E., Chien C.-C., Paster B.J., Ericson R.L., Orcutt R.P., Schauer D.B., Fox J.G. (1999). Phylogeny of the Defined Murine Microbiota: Altered Schaedler Flora. Appl. Environ. Microbiol..

[63] Aller M.-A. (2013). Splanchnic-Aortic Inflammatory Axis in Experimental Portal Hypertension. WJG.

[64] Sturm L., Hirose M., Stolz L., Schultheiss M., Zoldan K., Reincke M., Huber J.P., Kaeser R., Boettler T., Thimme R. (2023). Proton Pump Inhibitor Treatment Aggravates Bacterial Translocation in Patients with Advanced Cirrhosis and Portal Hypertension. mBio.

[65] Bellot P., García-Pagán J.C., Francés R., Abraldes J.G., Navasa M., Pérez-Mateo M., Such J., Bosch J. (2010). Bacterial DNA Translocation Is Associated with Systemic Circulatory Abnormalities and Intrahepatic Endothelial Dysfunction in Patients with Cirrhosis. Hepatology.

[66] Sersté T., Bourgeois N., Lebrec D., Evrard S., Devière J., Le Moine O. (2006). Relationship between the Degree of Portal Hypertension and the Onset of Spontaneous Bacterial Peritonitis in Patients with Cirrhosis. Acta Gastroenterol. Belg..

[67] Santopaolo F., Coppola G., Giuli L., Gasbarrini A., Ponziani F.R. (2022). Influence of Gut–Liver Axis on Portal Hypertension in Advanced Chronic Liver Disease: The Gut Microbiome as a New Protagonist in Therapeutic Management. Microbiol. Res..

[68] Geerts A.M., De Vriese A.S., Vanheule E., Van Vlierberghe H., Mortier S., Cheung K., Demetter P., Lameire N., De Vos M., Colle I. (2006). Increased Angiogenesis and Permeability in the Mesenteric Microvasculature of Rats with Cirrhosis and Portal Hypertension: An in Vivo Study. Liver Int..

[69] Shah A., Shanahan E., Macdonald G.A., Fletcher L., Ghasemi P., Morrison M., Jones M., Holtmann G. (2017). Systematic Review and Meta-Analysis: Prevalence of Small Intestinal Bacterial Overgrowth in Chronic Liver Disease. Semin. Liver Dis..

[70] Qin N., Yang F., Li A., Prifti E., Chen Y., Shao L., Guo J., Le Chatelier E., Yao J., Wu L. (2014). Alterations of the Human Gut Microbiome in Liver Cirrhosis. Nature.

[71] Bajaj J.S., Heuman D.M., Hylemon P.B., Sanyal A.J., White M.B., Monteith P., Noble N.A., Unser A.B., Daita K., Fisher A.R. (2014). Altered Profile of Human Gut Microbiome Is Associated with Cirrhosis and Its Complications. J. Hepatol..

[72] Bajaj J.S., Idilman R., Mabudian L., Hood M., Fagan A., Turan D., White M.B., Karakaya F., Wang J., Atalay R. (2018). Diet Affects Gut Microbiota and Modulates Hospitalization Risk Differentially in an International Cirrhosis Cohort. Hepatology.

[73] Chesta J., Defilippi C., Defilippi C. (1993). Abnormalities in Proximal Small Bowel Motility in Patients with Cirrhosis. Hepatology.

[74] Sadik R., Abrahamsson H., Björnsson E., Gunnarsdottir A., Stotzer P.-O. (2003). Etiology of Portal Hypertension May Influence Gastrointestinal Transit. Scand. J. Gastroenterol..

[75] Gunnarsdottir S.A., Sadik R., Shev S., Simrén M., Sjövall H., Stotzer P.-O., Abrahamsson H., Olsson R., Björnsson E.S. (2003). Small Intestinal Motility Disturbances and Bacterial Overgrowth in Patients with Liver Cirrhosis and Portal Hypertension. Am. J. Gastroenterol..

[76] Lorenzo-Zúñiga V., Bartolí R., Planas R., Hofmann A.F., Viñado B., Hagey L.R., Hernández J.M., Mañé J., Alvarez M.A., Ausina V. (2003). Oral Bile Acids Reduce Bacterial Overgrowth, Bacterial Translocation, and Endotoxemia in Cirrhotic Rats. Hepatology.

[77] Kakiyama G., Pandak W.M., Gillevet P.M., Hylemon P.B., Heuman D.M., Daita K., Takei H., Muto A., Nittono H., Ridlon J.M. (2013). Modulation of the Fecal Bile Acid Profile by Gut Microbiota in Cirrhosis. J. Hepatol..

[78] Kakiyama G., Hylemon P.B., Zhou H., Pandak W.M., Heuman D.M., Kang D.J., Takei H., Nittono H., Ridlon J.M., Fuchs M. (2014). Colonic Inflammation and Secondary Bile Acids in Alcoholic Cirrhosis. Am. J. Physiol.-Gastrointest. Liver Physiol..

[79] Albillos A., Abreu L., Alvarez-Mon M., Gea F., Gonzalo M.A., Rossi I., Garrido A., Barrios C., Escartin P. (1988). Study of the Secretion of Pepsinogen I in Cirrhotic Humans with and without Portacaval Shunt. Am. J. Gastroenterol..

[80] Shindo K., Machida M., Miyakawa K., Fukumura M. (1993). A Syndrome of Cirrhosis, Achlorhydria, Small Intestinal Bacterial Overgrowth, and Fat Malabsorption. Am. J. Gastroenterol..

[81] Llorente C., Jepsen P., Inamine T., Wang L., Bluemel S., Wang H.J., Loomba R., Bajaj J.S., Schubert M.L., Sikaroodi M. (2017). Gastric Acid Suppression Promotes Alcoholic Liver Disease by Inducing Overgrowth of Intestinal Enterococcus. Nat. Commun..

[82] Chen Y., Yang F., Lu H., Wang B., Chen Y., Lei D., Wang Y., Zhu B., Li L. (2011). Characterization of Fecal Microbial Communities in Patients with Liver Cirrhosis. Hepatology.

[83] Bajaj J.S., Hylemon P.B., Ridlon J.M., Heuman D.M., Daita K., White M.B., Monteith P., Noble N.A., Sikaroodi M., Gillevet P.M. (2012). Colonic Mucosal Microbiome Differs from Stool Microbiome in Cirrhosis and Hepatic Encephalopathy and Is Linked to Cognition and Inflammation. Am. J. Physiol.-Gastrointest. Liver Physiol..

[84] Pijls K.E., Jonkers D.M.A.E., Elamin E.E., Masclee A.A.M., Koek G.H. (2013). Intestinal Epithelial Barrier Function in Liver Cirrhosis: An Extensive Review of the Literature. Liver Int..

[85] Albillos A., Lario M., Álvarez-Mon M. (2014). Cirrhosis-Associated Immune Dysfunction: Distinctive Features and Clinical Relevance. J. Hepatol..

[86] Muñoz L., José Borrero M., Ubeda M., Lario M., Díaz D., Francés R., Monserrat J., Pastor O., Aguado-Fraile E., Such J. (2012). Interaction between Intestinal Dendritic Cells and Bacteria Translocated from the Gut in Rats with Cirrhosis. Hepatology.

[87] Garcia-Tsao G., Lee F.-Y., Barden G.E., Cartun R., Brian West A. (1995). Bacterial Translocation to Mesenteric Lymph Nodes Is Increased in Cirrhotic Rats with Ascites. Gastroenterology.

[88] Wiest R., Lawson M., Geuking M. (2014). Pathological Bacterial Translocation in Liver Cirrhosis. J. Hepatol..

[89] Pérez-Paramo M., Munoz J., Albillos A., Freile I., Portero F., Santos M., Ortiz-Berrocal J. (2000). Effect of Propranolol on the Factors Promoting Bacterial Translocation in Cirrhotic Rats with Ascites. Hepatology.

[90] Schwabl P., Hambruch E., Seeland B.A., Hayden H., Wagner M., Garnys L., Strobel B., Schubert T.-L., Riedl F., Mitteregger D. (2017). The FXR Agonist PX20606 Ameliorates Portal Hypertension by Targeting Vascular Remodelling and Sinusoidal Dysfunction. J. Hepatol..

[91] Verbeke L., Farre R., Verbinnen B., Covens K., Vanuytsel T., Verhaegen J., Komuta M., Roskams T., Chatterjee S., Annaert P. (2015). The FXR Agonist Obeticholic Acid Prevents Gut Barrier Dysfunction and Bacterial Translocation in Cholestatic Rats. Am. J. Pathol..

[92] Inagaki T., Moschetta A., Lee Y.-K., Peng L., Zhao G., Downes M., Yu R.T., Shelton J.M., Richardson J.A., Repa J.J. (2006). Regulation of Antibacterial Defense in the Small Intestine by the Nuclear Bile Acid Receptor. Proc. Natl. Acad. Sci. USA.

[93] Úbeda M., Lario M., Muñoz L., Borrero M.-J., Rodríguez-Serrano M., Sánchez-Díaz A.-M., Del Campo R., Lledó L., Pastor Ó., García-Bermejo L. (2016). Obeticholic Acid Reduces Bacterial Translocation and Inhibits Intestinal Inflammation in Cirrhotic Rats. J. Hepatol..

[94] Llovet J.M., Bartolí R., Planas R., Cabré E., Jimenez M., Urban A., Ojanguren I., Arnal J., Gassull M.A. (1994). Bacterial Translocation in Cirrhotic Rats. Its Role in the Development of Spontaneous Bacterial Peritonitis. Gut.

[95] Du Plessis J., Vanheel H., Janssen C.E.I., Roos L., Slavik T., Stivaktas P.I., Nieuwoudt M., Van Wyk S.G., Vieira W., Pretorius E. (2013). Activated Intestinal Macrophages in Patients with Cirrhosis Release NO and IL-6 That May Disrupt Intestinal Barrier Function. J. Hepatol..

[96] Shi H., Lv L., Cao H., Lu H., Zhou N., Yang J., Jiang H., Dong H., Hu X., Yu W. (2017). Bacterial Translocation Aggravates CCl4-Induced Liver Cirrhosis by Regulating CD4+ T Cells in Rats. Sci. Rep..

[97] Le Roy T., Llopis M., Lepage P., Bruneau A., Rabot S., Bevilacqua C., Martin P., Philippe C., Walker F., Bado A. (2013). Intestinal Microbiota Determines Development of Non-Alcoholic Fatty Liver Disease in Mice. Gut.

[98] De Minicis S., Rychlicki C., Agostinelli L., Saccomanno S., Candelaresi C., Trozzi L., Mingarelli E., Facinelli B., Magi G., Palmieri C. (2014). Dysbiosis Contributes to Fibrogenesis in the Course of Chronic Liver Injury in Mice. Hepatology.

